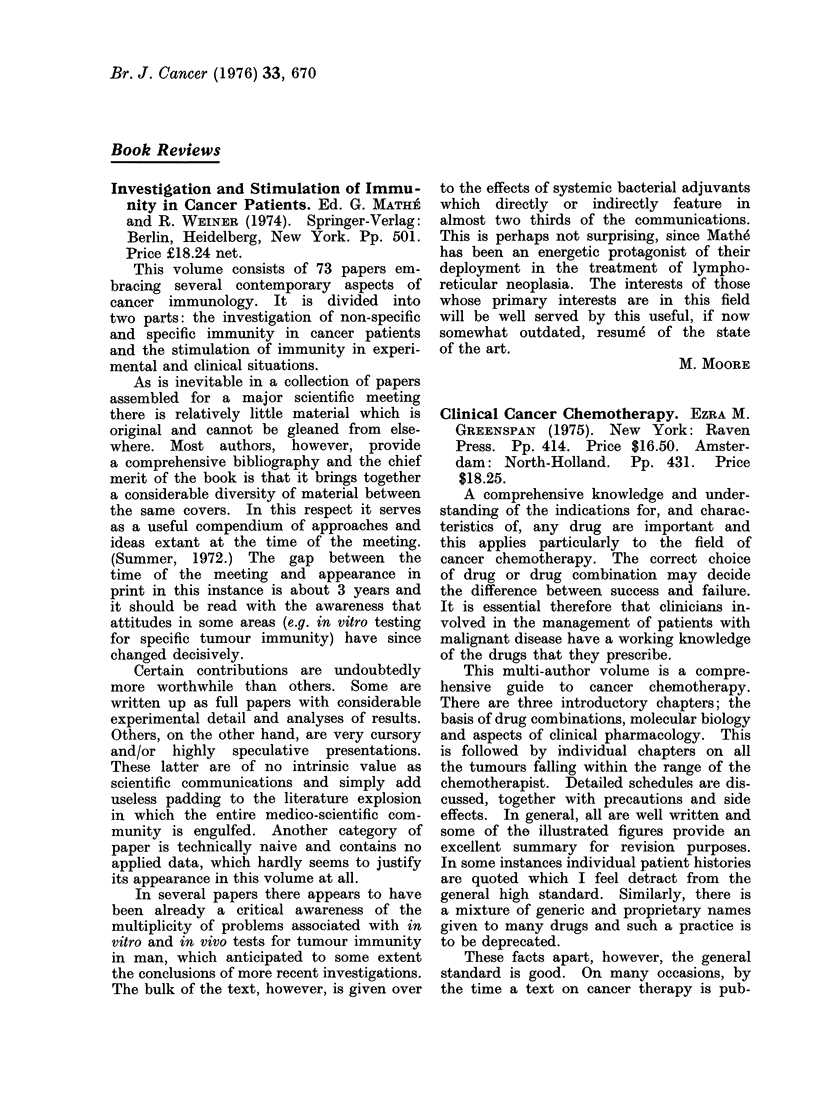# Investigation and Stimulation of Immunity in Cancer Patients

**Published:** 1976-06

**Authors:** M. Moore


### *Book Reviews*

**Investigation and Stimulation of Immunity in Cancer Patients.** Ed. G. Mathé and R. Weiner (1974). Springer-Verlag: Berlin, Heidelberg, New York. Pp. 501. Price £18.24 net.

This volume consists of 73 papers embracing several contemporary aspects of cancer immunology. It is divided into two parts: the investigation of non-specific and specific immunity in cancer patients and the stimulation of immunity in experimental and clinical situations.

As is inevitable in a collection of papers assembled for a major scientific meeting there is relatively little material which is original and cannot be gleaned from elsewhere. Most authors, however, provide a comprehensive bibliography and the chief merit of the book is that it brings together a considerable diversity of material between the same covers. In this respect it serves as a useful compendium of approaches and ideas extant at the time of the meeting. (Summer, 1972.) The gap between the time of the meeting and appearance in print in this instance is about 3 years and it should be read with the awareness that attitudes in some areas (*e.g. in vitro* testing for specific tumour immunity) have since changed decisively.

Certain contributions are undoubtedly more worthwhile than others. Some are written up as full papers with considerable experimental detail and analyses of results. Others, on the other hand, are very cursory and/or highly speculative presentations. These latter are of no intrinsic value as scientific communications and simply add useless padding to the literature explosion in which the entire medico-scientific community is engulfed. Another category of paper is technically naive and contains no applied data, which hardly seems to justify its appearance in this volume at all.

In several papers there appears to have been already a critical awareness of the multiplicity of problems associated with *in vitro* and *in vivo* tests for tumour immunity in man, which anticipated to some extent the conclusions of more recent investigations. The bulk of the text, however, is given over to the effects of systemic bacterial adjuvants which directly or indirectly feature in almost two thirds of the communications. This is perhaps not surprising, since Mathé has been an energetic protagonist of their deployment in the treatment of lymphoreticular neoplasia. The interests of those whose primary interests are in this field will be well served by this useful, if now somewhat outdated, resumé of the state of the art.

M. Moore